# The Effect of Nutrition Intervention with Oral Nutritional Supplements on Pancreatic and Bile Duct Cancer Patients Undergoing Chemotherapy

**DOI:** 10.3390/nu11051145

**Published:** 2019-05-22

**Authors:** Seong Hyeon Kim, Song Mi Lee, Hei Cheul Jeung, Ik Jae Lee, Joon Seong Park, Mina Song, Dong Ki Lee, Seung-Min Lee

**Affiliations:** 1 Clinical Nutrition Program, Graduate School of Human Environmental Sciences, Yonsei University, Seoul 03722, Korea; cca0926@hanmail.net; 2 Department of Nutrition Care, Severance Hospital, Yonsei University College of Medicine, Seoul 03722, Korea; NUTRPINE@yuhs.ac; 3 Cancer Metastasis Research Center, Division of Medical Oncology, Cancer Center Gangnam Severance Hospital, Yonsei University College of Medicine, Seoul 06273, Korea; jeunghc1123@yuhs.ac; 4 Department of Radiation Oncology, Gangnam Severance Hospital, Yonsei University College of Medicine, Seoul 06273, Korea; ikjae412@yuhs.ac; 5 Pancreatobiliary Cancer Clinic, Department of Surgery, Gangnam Severance Hospital, Yonsei University College of Medicine, Seoul 06273, Korea; jspark330@yuhs.ac; 6 Department of Food and Nutrition, Brain Korea 21 PLUS Project, College of Human Ecology, Yonsei University, Seoul 03722, Korea; msyu@yonsei.ac.kr; 7 Department of Internal Medicine, Gangnam Severance Hospital, Yonsei University College of Medicine, Seoul 06273, Korea

**Keywords:** pancreatic and bile duct cancer, chemotherapy, oral nutritional supplement (ONS), nutritional status, patient-generated subjective global assessments, Quality of Life Questionnaire Core 30

## Abstract

Chemotherapy may negatively affect nutritional status and quality of life (QOL) in pancreatic cancer patients. Our aim was to investigate the beneficial effects of oral nutrition supplements (ONS) on pancreatic and bile duct cancer patients undergoing chemotherapy. Among patients with progressive pancreatic and bile duct cancer receiving chemotherapy, the ONS group (*n* = 15) received two packs of ONS daily for 8 weeks while the non-ONS group (*n* = 19) did not. Anthropometric measures, dietary intake, nutritional status, and quality of life were assessed. ONS significantly increased daily intakes of energy, carbohydrates, proteins, and lipids at 8 weeks compared to the baseline. After 8 weeks, fat mass significantly increased in the ONS group. For patients in their first cycle of chemotherapy, body weight, fat-free mass, skeletal muscle mass, body cell mass, and fat mass increased in the ONS group but decreased in the non-ONS group. Fat mass increased in second or higher cycle only in the ONS group. Patient-generated subjective global assessments (PG-SGA) and fatigue scores in the Quality of Life Questionnaire Core 30 (QLQ-C30) improved in the ONS group. ONS might improve nutritional status by increasing fat mass and/or maintaining the body composition of pancreatic and bile duct cancer patients with chemotherapy, especially those in the first cycle, and alleviate fatigue symptoms.

## 1. Introduction

Pancreatic cancer is the 13th most common cancer, and its mortality rate ranks eighth among all cancer types [1]. Chemotherapy is used as part of the treatment of patients with stage III (regional progress) and stage IV (metastasis) pancreatic cancers which are categorized as inoperable by the American Joint Committee on Cancer (AJCC) [2]. The high recurrence rate of pancreatic cancer after operation also increases the frequency of treatment with chemotherapy, even when discovered at an early stage [3]. Despite the advantages of chemotherapy, it causes diverse nutritional side effects due to its non-selective action on normal cells. These include loss of appetite, nausea, vomiting, and stomatitis, which can disturb oral food intake and digestive nutrient absorption [4], which accompany weight loss, decreased anticancer drug reaction rate, increased anticancer drug toxicity [5], reduced survival rate [6], and reduced quality of life (QoL) [7]. Pancreatic and bile duct cancer shows the second-highest malnutrition rate (47.6%) [8]. Thus, constant monitoring and early nutritional intervention are crucial for the prevention of malnutrition in cancer patients receiving chemotherapy [9].

The European Society for Clinical Nutrition and Metabolism (ESPEN) and guidelines in Australia, Europe, Great Britain, and the US recommend oral nutritional supplement (ONS) provision during treatment to increase oral intake in malnourished cancer patients [10,11,12,13]. ONSs are convenient, ready-made products that contain balanced nutrients, calories, and proteins to complement insufficient oral intake [14]. ONS supply was shown to be beneficial in increasing oral intake and body weight, maintaining fat-free mass and increasing the QoL in pancreatic cancer patients [15,16]. However, current studies have focused on weight and lean body mass [15,16,17,18]. One study on gastrointestinal and lung cancer patients treated with palliative chemotherapy, including pancreatic cancer patients, showed no significant improvement in survival rate and QoL regardless of nutritional interventions (nutritional supplements, vitamins or dietary advice) [19]. Research on the effect of ONS on pancreatic and bile duct cancer patients receiving chemotherapy is further required.

The nutritional statuses of cancer patients can be evaluated by the patient-generated subjective global assessment (PG-SGA), which provides information equivalent to objective indexes such as the patient’s disease history and dietary history (weight change, food intake, two or more weeks of continued gastrointestinal abnormality and physical function), as well as to clinical tests (body fat loss, muscle mass loss, existence of edema and hydrops abdominis) [20]. Higher scores (≥9) reflect higher risks of malnutrition [20]. It has been recommended and used to assess the nutritional statuses of pancreatic and bile duct cancer patients with early postoperative enteral feeding [21]. In a cross-sectional study, the mean PG-SGA score of pancreatic and bile duct cancer patients was 10.94, which is higher than that of the colon (9.54) or rectal cancer (4.67), and similar to gastric cancer (10.35) [22].

The measurement of QoL has been associated with patients’ prognoses and has been effectively used in clinical trials to examine cancer patients [23]. Barber et al. found that ONS in pancreatic cancer patients alleviated the loss of appetite and improved overall performance status [15]. The overall QoL improved in patients whose weight increased [17]. The European Organization for Research and Treatment of Cancer Quality of Life Questionnaire Core30 (EORTC QLQ-C30) is a tool for evaluating the QoL of cancer patients which includes five functional scales (physical, role, emotional, cognitive and social scales), symptom scales and a global scale [23].

In the current study, we investigated the effects of ONS on pancreatic and bile duct cancer patients undergoing chemotherapy by examining changes in nutritional status (weight, body composition and PG-SGA), nutritional intake and QoL with EORTC QLQ-C30.

## 2. Materials and Methods

### 2.1. Study Population

This study proceeded according to the Declaration of Helsinki and was approved by the Institutional Review Board of the Gangnam Severance Hospital (project number: 3-2015-0033). From April 2015 to October 2016, we enrolled patients from the hospital aged older than 20 years who were diagnosed with progressive (metastatic) pancreatic and bile duct cancer and were scheduled to receive chemotherapy. All subjects signed an informed consent before participating in the study. Patients with liver failure (over a two-fold increase in aspartate transaminase/alanine transaminase), kidney failure (over a two-fold increase in blood urea nitrogen/creatinine), severe seroperitoneum or edema affecting weight evaluation, cancer that spread to the brain, body mass index (BMI) > 30 kg/m^2^, patients incapable of oral intake, patients who had recently undergone pancreatic and bile duct cancer operation that could affect weight evaluation due to postsurgical digestive problems, and illiterate and foreign patients were excluded.

**Figure 1:**
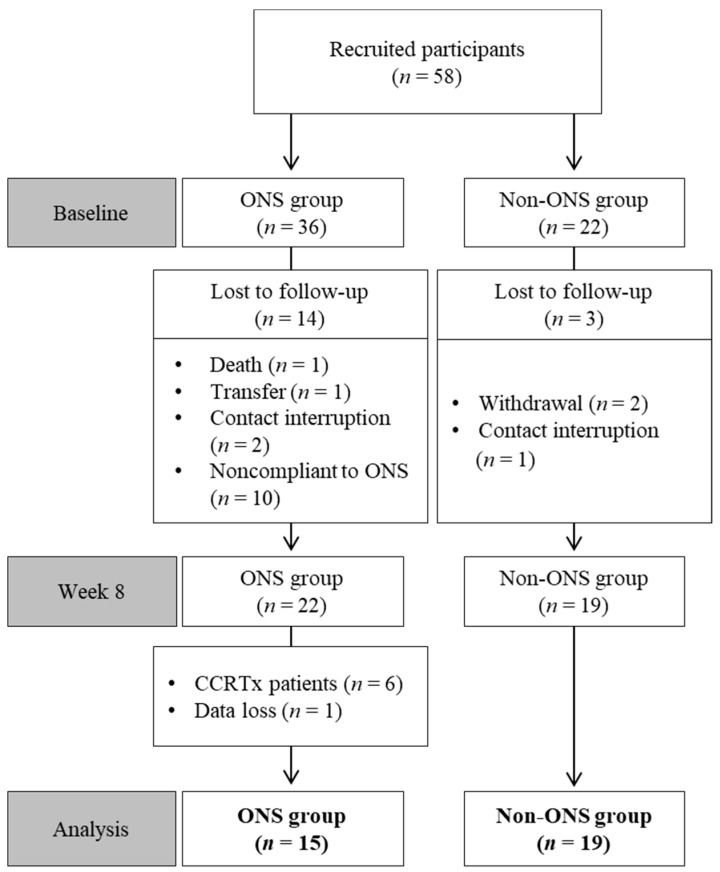
Flow chart of patient enrollment, randomization and exclusion. CCRTx: radiochemotherapy; ONS: oral nutritional supplement.

Among 58 enrolled patients, 34 participants were included in the final analysis after excluding those who dropped out or had missing data (Table 1). Reasons for drop-out included death, treatment withdrawal, transfer, treatment change to radiochemotherapy, contact interruption and ONS compliance under 1.5 packs per day (Figure 1).

### 2.2. Study Design

In this prospective study, patients were randomly assigned either to the ONS or non-ONS groups via a simple randomization using the Statistical Analysis Software (SAS) 9.4 (SAS Institute Inc., Cary, North Carolina, U.S.). In the ONS group, two ONS packs were administered daily in addition to regular meals, and ONS intake was checked at every interview session. Except for ONS provision, the non-ONS and ONS groups received the same nutritional care. Dietetic therapy for pancreatic and bile duct cancer was administered in week 1. In week 2, patients were advised of the management of eating-related side effects from chemotherapy. In week 4, they received education on high-calorie and high-protein diets. In week 8, the same indexes used in the baseline evaluations were reevaluated. A trained dietitian evaluated all patients in terms of their anthropometry, PG-SGA, bioelectrical impedance analysis (BIA), and QoL measurements in the 1st and 8th weeks. Dietary intake and body weight were assessed in weeks 1, 2, 4, and 8. Data regarding the patients’ nutritional statuses, medical history and biochemical test results were collected from electronic medical records.

### 2.3. Oral Nutritional Supplements

The ONS used in this study was the Medifood Miniwell OS (Korea Medical Food, Seoul, Korea), which is a balanced nourishing food that contains 1.33 kcal/mL. This product was developed to improve the oral intake of patients with a declined appetite and digestive function. Miniwell OS contains 200 kcal energy, 9 g protein, 6 g fat, 29 g carbohydrate (P: F: C = 17.5: 26.2: 56.3, % kcal) and 2.5 g fiber per pack (150 mL). The expected daily nutritional intake in the ONS group was 400 kcal energy, 19 g protein, 12 g fat and 58 g carbohydrate per two ONS packs.

### 2.4. Investigation of General Details and Anthropometry

Data regarding sex, age, height (cm), weight (kg), BMI, diagnosis and medical history of diseases were collected from medical records. Variables related to chemotherapy such as the administration dates and administration details were also investigated. The weight (kg) at the time of hospitalization was measured, and weight history was checked by examining the patients’ normal weight through interview (for patients starting chemotherapy: the average weight before diagnosis; for patients restarting chemotherapy: the average weight before the start of the recent treatment).

### 2.5. Dietary Intake Evaluation

All subjects underwent four sessions of individual nutritional consultation with a trained dietitian over the eight weeks of the research period. For the ONS group, the amount of daily ONS intake was recorded and checked by the dietitian during the consultation sessions. For both groups, regular meal and snack (including ONS) intake data were first collected through the patients’ self-recorded daily food intake record sheets using the 24-h recall method. Then the dietitian interviewed each patient using food models or replicas to confirm and evaluate their accurate intake. Finally, dietary intake was assessed based on calories and three major nutrients (carbohydrates, protein, lipids) in grams using CAN-Pro 4.0 (The Korean Nutrition Society, Seoul, Korea). The recommended calorie intake was set at 30 kcal/kg and protein at 1.2 g/kg [10].

### 2.6. Body Composition Analysis

Body composition analysis was performed using the multi-frequency impedance method with INBODY S10 (Biospace, Seoul, Korea). BIA results were recorded by the dietitian at the start (week 0) and end (week 8) of the study. Fat-free mass (kg), skeletal muscle mass (kg), body cell mass (kg), and fat mass (kg) were evaluated and analyzed.

### 2.7. Evaluation of Nutritional Status and Health-Related Quality of Life

PG-SGA was used as a prognostic tool to evaluate the nutritional statuses of patients with cancer [20]. It included questions related to nutritional symptoms, short-term weight loss, and other medical history [20]. The total score was compared and analyzed in three groups: A, well-nourished; B, moderately or suspected of being malnourished; C, severely malnourished [20]. Health-related QoL is defined as the subjective level of satisfaction with one’s current physical, mental, social, and cognitive functions, compared with the ideal status of those functions as recognized by the subject [24]. The present study used the Korean translation of the Quality of Life Questionnaire Core 30 (QLQ-C30) developed by the European Organization for Research and Treatment of Cancer (EORTC) as the measurement tool for health-related QoL. The EORTC QLQ-C30 comprises 30 questions organized into three subcategories: overall QoL, functional scales, and symptomatic scales. The responses are converted based on the scoring manual of the EORTC QLQ-C30 (version 3.0), and QoL is considered higher when overall QoL and functional scores are higher and symptomatic score is lower [25].

### 2.8. Biochemical Tests

For the biochemical tests, the following data were gathered from the hospital electronic medical records: serum albumin (g/dL), serum total protein (g/dL), serum total cholesterol (mg/dL), total lymphocyte count (×103/μL), absolute neutrophil count (×103/μL), hemoglobin (g/dL) and hematocrit (%). The biochemical test results obtained within one week of the interview were used.

### 2.9. Statistical Analysis

The results were analyzed using SPSS Statistics version 23.0 (IBM Corp., Armonk, NY, USA) and were reported as mean and standard errors or frequencies and percentages. Fisher’s exact test was used to assess categorical variables such as sex, diagnosis, type of chemotherapy and type of drugs, whereas the McNemar test was used to calculate the changes within groups for categorical variables with scales such as the PG-SGA. For continuous variables such as anthropometry, body composition, nutrient intake quantity, PG-SGA scores and QoL evaluation scores, nonparametric statistical methods such as the Wilcoxon signed-rank test and the Mann–Whitney U test were used to supplement the small number of participants. Statistical significance was verified at a p-value of <0.05.

## 3. Results

### 3.1. General Details

The subjects were 65.2 years old on average and received either adjuvant chemotherapy (23.5%) or palliative chemotherapy (76.5%) (Table 1). About 61.8% of the subjects were in the first cycle of chemotherapy at the time of study participation, and the rest were in their second or higher cycle of chemotherapy. The non-ONS and ONS groups demonstrated no significant differences in terms of age, the ratio of males to females, diagnosis, clinical stage, type of chemotherapy, type of anti-cancer medicine, starting date of chemotherapy, BMI, recommended energy allowance (in kcal) or recommended protein allowance. Cancer progression throughout the study period at week 8 compared with baseline is shown in Supplementary Table S1.

### 3.2. Analyses of Calories Absorbed through Oral Intake and Nutrients

**Figure 2:**
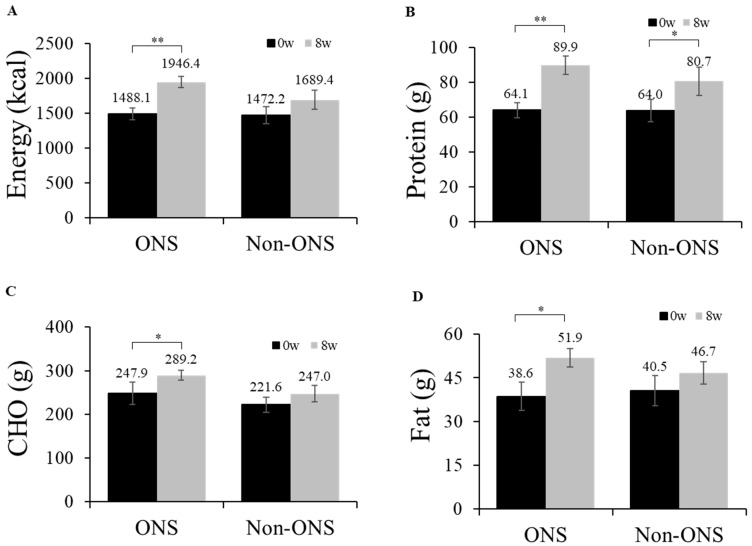
Daily oral intake at week 0 and week 8. Changes in energy intake (**A**), protein intake (**B**), carbohydrate intake (**C**), and fat intake (**D**) are shown. Values are mean ± SE. Significant differences between the ONS group and non-ONS group were analyzed by the Mann–Whitney test. Significant differences between 0 weeks (baseline) and 8 weeks were determined by the Wilcoxon signed rank test. *CHO* carbohydrate, * *p* < 0.05; ** *p* < 0.01.

The ONS group showed significant increases in its dietary intakes of calories (*p* = 0.001), proteins (*p* = 0.001), carbohydrates (*p* = 0.015) and lipids (*p* = 0.023) at 8 weeks compared to the baseline (Figure 2). However, the non-ONS group did not show significant increases except for protein intake (*p* = 0.040) (Figure 2). When the change values of dietary intake between 0 and 8 weeks were compared, there were no significant differences between the non-ONS and ONS groups. Considering 100% compliance to ONS as 2 packs/day (300mL, 400kcal), the total average compliance was 90.2% (data not shown). The intakes of regular meal intake and snack, including ONS, are detailed in Supplementary Table S2.

### 3.3. Analysis of Change in Body Composition

**Figure 3:**
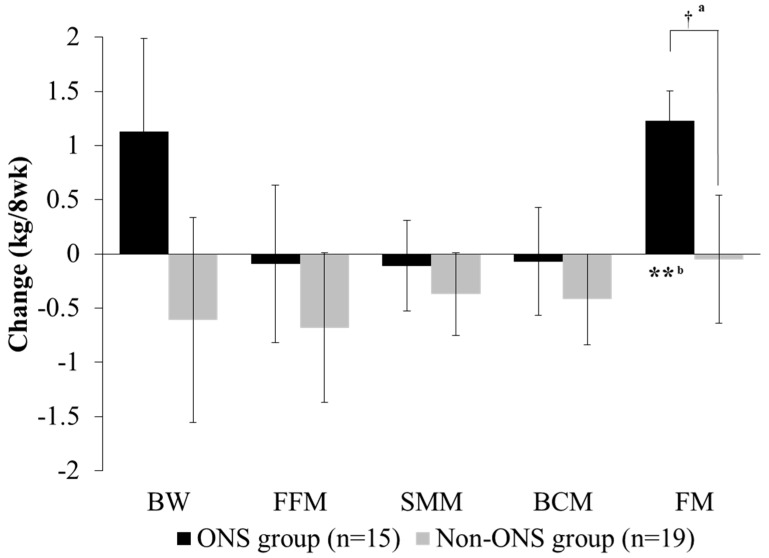
Change of body composition between groups. Values are mean. Change value = (8-week data) − (0-week data). ^a^ Significant differences between the ONS group and non-ONS group were derived by the Mann–Whitney test (†: *P* < 0.05). ^b^ Significant differences between 0 weeks (baseline) and 8 weeks were derived by the Wilcoxon signed rank test (**: *P* < 0.01). BW, body weight; FFM, fat-free mass; SMM, skeletal muscle mass; BCM: body cell mass; FM: fat mass.

Both the non-ONS and ONS groups did not show significant changes in body weight, fat-free mass, skeletal muscle mass and body cell mass at 8 weeks compared to the baseline (Figure 3). However, fat mass was significantly increased in the ONS group after 8 weeks of intervention, whereas there was no difference in the non-ONS group. The change values between 0 and 8 weeks for fat mass were also significantly different between the ONS group and non-ONS group.

Because the duration of chemotherapy treatment may affect body weight [26], we further subdivided the non-ONS and ONS groups based on the number of cycles of chemotherapy (Table 2). The ONS group showed increased body weight and maintained fat-free mass, skeletal muscle mass, body cell mass and fat mass in patients undergoing the first cycle of chemotherapy, and increased fat mass in those undergoing their second or higher cycle of chemotherapy. The non-ONS group showed negative values in all body composition variables except fat mass in patients in their first cycle of chemotherapy and no changes in those in their second or higher cycle of chemotherapy.

### 3.4. Analysis of PG-SGA Score and Grade

**Figure 4:**
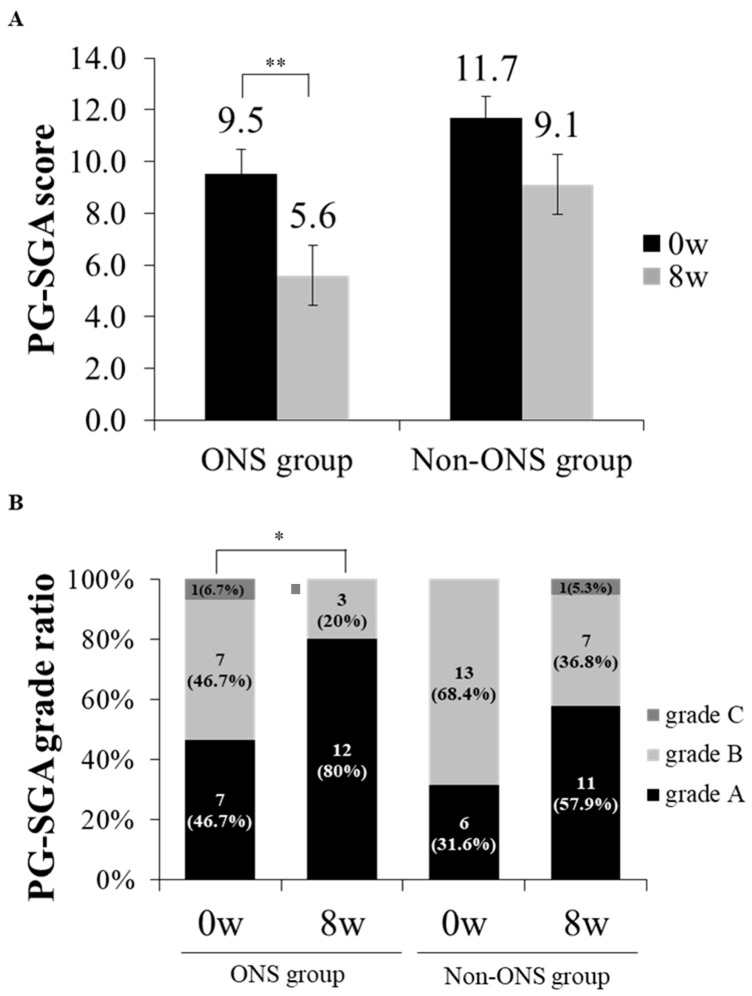
The change of patient-generated subjective global assessment (PG-SGA) scores and grade ratios between ONS and non-ONS groups. A comparison of PG-SGA scores between 0 and 8 weeks are shown for each group (**A**). A comparison of PG-SGA by the ratio of participants in each grade between 0 and 8 weeks are shown for each group (**B**). Significant differences in the PG-SGA scores between 0 weeks and 8 weeks in the ONS group were derived by the Wilcoxon signed rank test (* *p* < 0.05; ** *p* < 0.01). Values are mean. Grade A: well-nourished; Grade B: moderately malnourished; Grade C: severely malnourished.

The PG-SGA score and grade which were used to evaluate nutritional status were compared between the non-ONS and ONS groups (Figure 4A,B). The ONS group lowered PG-SGA scores from 9.5 ± 0.9 to 5.6 ± 0.8 (*p* = 0.002) at 8 weeks after intervention, while the non-ONS group did not change in this regard (Figure 3A). On the other hand, the ONS and non-ONS groups showed significant changes in PG-SGA grades with increases in grade A (46.7% to 80% and 31.6% to 57.9%, respectively) (Figure 4B). These data indicate that both the ONS and non-ONS groups improved their nutritional statuses at the end of the study, but the ONS group appeared to be more effective in terms of the PG-SGA score.

### 3.5. Analysis of Health-Related Quality of Life

The results of the health-related QoL analysis using the EORTC QLQ-C30 (ver. 3.0) are presented in Table 3. In both groups, the overall QoL score marginally improved at 8 weeks compared to the baseline but was not significant (Table 3). In the symptomatic scales category, fatigue was significantly decreased at 8 weeks in the ONS group compared to the baseline, and pain was significantly decreased in the non-ONS group (Table 3). No significant changes were detected in the functional scales (Table 3). None of the biochemical tests for total protein, total cholesterol, absolute neutrophil count, total lymphocyte count, hemoglobin and hematocrit showed significant differences after 8 weeks compared to the baseline in both groups, and the change values were not different between the two groups, except albumin (Table 4).

## 4. Discussion

Cancer patients receiving chemotherapy require sufficient nutritional intervention to maintain body weight for a better prognosis because chemotherapy increases their risk of malnutrition and weight loss [9]. In our study, the ONS and non-ONS groups received the same nutritional support except for the supply of ONS, which involved education to increase total oral intake by consuming foods with high nutrient density, and by increasing the frequencies of meals and/or snacks. We hypothesized that the use of ONS could aid the maintenance of nutritional status in patients who had started and/or were undergoing chemotherapy. We demonstrated that ONS indeed increased fat mass in pancreatic and bile duct cancer patients receiving chemotherapy along with an improvement of the nutritional status score and fatigue symptom score in the EORTC QLQ-C30. These effects were especially distinctive in the first cycle of chemotherapy.

During the study period, there were no significantly different changes in the dietary intake between the non-ONS and ONS groups, although there were increases after 8 weeks in the dietary intakes of calories, carbohydrates, and lipids only in the ONS group. These could be due to a compensatory reduction in meal intake in the ONS group, causing a lack of a significant increase compared to the non-ONS group. However, the ONS group recorded a significant increase in fat mass, which was not seen in the non-ONS group. About 90% of the total energy source of the body is fat, which contributes to the maintenance of weight and nutritional status in cachectic cancer patients (fat loss causes weight loss in cachectic cancer patients) [27]. The reduction in body fat in cachectic patients is due to abnormal enzymatic activation (caused by cachexia) that decreases lipogenesis while increasing lipolysis [28]. When approximately 80% of body fat is depleted, the total body weight is reduced by 30%, which causes death [29]. In progressive cancer patients, a higher rate of fat mass reduction results in a significant decrease in the survival rate [30]. The absolute gain of visceral adipose tissue is associated with better overall survival and progression-free survival rates in pancreatic cancer patients treated with chemotherapy and radiochemotherapy [31]. Thus, the gaining of fat mass by the ONS group might contribute to good prognosis in pancreatic and bile duct cancer patients receiving chemotherapy.

Previous studies on ONS demonstrated that cancer patients receiving ONS, for 6 months [32] and 3 months [33], increased their body weight and fat mass. However, in this study, ONS did not significantly change the weights of pancreatic and bile duct cancer patients over 8 weeks. Considering a previous report on a positive correlation between the change in body weight and body fat mass during chemotherapy [26], an increase in fat mass seen in our study might have led to an increase in body weight if we had prolonged the study for longer than 8 weeks.

The different timing of ONS during the treatment of chemotherapy might affect the effects of ONS due to different nutritional statuses at the starting time of ONS. In order to clarify the effects of ONS on the changes in body composition, we subdivided the subjects according to their cycles of chemotherapy. The ONS effects were greater in those starting chemotherapy when provided with ONS than those who were already receiving chemotherapy. All parameters of body composition, including body weight, skeletal muscle mass, and fat mass were increased significantly among patients in their first cycle on ONS, while only fat mass was increased among those in their second or higher cycle of chemotherapy. On the other hand, those without ONS treatment lost body weight, fat-free mass and skeletal muscle mass, except fat mass, when they were in their first cycle of chemotherapy, and ONS was ineffective among those in their second or higher cycle of chemotherapy. The results of the body composition change in patients in their first cycle of chemotherapy are consistent with previous studies which found that enriched oral supplements preserved lean body mass and body weight in cancer patients [15,17,34,35,36]. Thus, our study suggests that ONS appears to be effective in the maintenance and/or increasing of body weight and skeletal muscle mass when the nutritional status has not yet been negatively affected by chemotherapy treatment at the starting time of ONS. Fat mass is positively influenced by ONS regardless of the cycle stages. This may suggest that the consideration of the timing of ONS supply is important to maximize the ONS effects for cancer patients who are scheduled to receive chemotherapy. Previous studies have also reported that nutritional intervention in the early stages of chemotherapy minimized the risk of malnutrition [37], increased the rate of chemotherapy completion [38] and improved the survival rate [39].

ONS appeared to be effective in improving the nutritional status of patients undergoing chemotherapy. Although the ONS and non-ONS groups both recorded improved nutritional statuses with respect to the PG-SGA grades, the ONS group alone significantly decreased PG-SGA scores from 9.5 ± 0.9 (baseline) to 5.6 ± 0.8 (week 8) compared to the non-ONS group. A PG-SGA score of 9 or higher in cancer patients has been shown to be associated with an 80% risk of malnutrition [6]. However, blood parameters for nutritional status were not significantly affected by ONS. This could be due to unstable metabolic conditions in the patients receiving chemotherapy. Similarly, a previous study for 4 weeks of ONS on esophageal cancer patients undergoing chemotherapy or radiation therapy reported no significant changes in the total serum protein, serum albumin, total cholesterol and hemoglobin [36].

When the QoL was assessed using the EORTC QLQ-C30 (ver. 3.0), the ONS-specific effect was detected only in fatigue in the symptomatic scales category. Fatigue is most commonly experienced by cancer patients during therapy and has a negative effect on daily activities, reducing the overall QoL [40]. ONS might have marginally improved the QoL, although not significantly, by decreasing fatigue. This is partially in accordance with previous reports in which ONS improved fatigue, and loss of appetite had been reported previously in cachectic cancer patients who underwent palliative treatments [32] and increased the overall QoL of unresectable pancreatic cancer patients [17].

There are several limitations of our study, including a high drop-out rate due to the poor prognosis of pancreatic and bile duct cancer and the small sample size within each group (ONS, *n* = 15; non-ONS, *n* = 19). Although this was not a double-blinded study, the collection and evaluation of data was strictly limited to a certified dietitian to minimize any observer’s bias. Despite such factors, we observed significant improvement in fat mass in the ONS group, as well as PG-SGA scores and fatigue compared to the non-ONS group, suggesting advantages of using ONS in patients receiving chemotherapy. Also, any effect of chemotherapy would have been detected in the non-ONS group between the baseline and week 8, and if it did, it would have been found in the non-ONS group as well. However, significant changes in pain and albumin that were seen in the non-ONS group did not appear significant in the ONS group and are difficult to explore as effects of chemotherapy.

In conclusion, apart from regular meals, the supply of ONS in pancreatic and bile duct cancer patients undergoing chemotherapy might promote their health by increasing body fat mass, improving PG-SGA scores and decreasing fatigue symptoms. In particular, patients receiving their first cycle of chemotherapy might benefit more from the use of ONS than those in their second or higher cycle of chemotherapy.

## Figures and Tables

**Table 1:** Baseline characteristics of patients.

Variable	ONS Group (*n* = 15)	Non-ONS Group (*n* = 19)	*P*-value ^e^
Sex (M:F) ^a^	7(46.7): 8(53.3)	9(47.4): 10(52.6)	1.000
Age (yrs) ^b^	64.5 ± 2.6	65.8 ± 2.1	0.584 ^f^
Major diagnosis			
Pancreatic cancer	3(20.0)	7(36.8)	0.451
Cholangiocellular carcinoma	1(6.7)	6(31.6)	
Common bile duct cancer	5(33.3)	5(26.3)	
Gallbladder cancer	6(40.0)	1(5.3)	
Stage			
II	5(33.3)	3(15.8)	0.066
III	0(0.0)	5(26.3)	
IV	10(66.7)	11(57.9)	
Type of chemotherapy			
adjuvant chemotherapy	4(26.7)	4(21.1)	1.000
palliative chemotherapy	11(73.3)	15(78.9)	
Chemotherapy regimen			
Gemcitabine	9(60.0)	16(84.2)	0.139
FL	6(40.0)	3(15.8)	
Variable	ONS group (*n* = 15)	Non-ONS group (*n* = 19)	*P*-value ^e^
Chemotherapy cycle			
1^st^	9(60.0)	12(63.2)	1.000
2^nd^ more	6(40.0)	7(36.8)	
BMI (kg/m^2^)	22.9 ± 0.6	23.5 ± 0.7	0.271 ^f^
Energy requirement (kcal/day) ^c^	1688.7 ± 50.5	1651.2 ± 52.6	0.410 ^f^
Protein requirement (g/day) ^d^	67.6 ± 2.0	67.7 ± 2.0	0.890 ^f^

*FL* 5-FU+leucovorin. ^a^ *n*(%); ^b^ Mean ± SE; ^c^ BMI < 25: Actual body weight × 30 kcal/day, BMI ≥ 25: Ideal body weight × 30 kcal/day; ^d^ BMI < 25: Actual body weight × 1.2 g/day, BMI ≥ 25: Ideal body weight × 1.2 g/day; ^e^ *P*-values calculated by Fisher’s test between the experimental group and control group; ^f^ *P*-values calculated by the Mann–Whitney test between the experimental group and control group.

**Table 2:** Change of body composition by chemotherapy cycle.

	ONS Group (*n* = 15)		Non-ONS Group (*n* = 19)				
	0 weeks	8 weeks	0 weeks	8 weeks	*p^a^*	*p^b^*	*p^c^*
1^st^ cycle							
BW (kg)	57.28 ± 2.2	59.32 ± 2.3	60.69 ± 2.5	59.83 ± 2.8	0.033	0.859	
Change	2.04 ± 0.7		−0.86 ± 1.2				0.049
FFM (kg)	43.73 ± 2.5	44.74 ± 2.5	42.96 ± 2.2	41.92 ± 2.3	0.058	0.169	
Change	1.01 ± 0.5		−1.04 ± 0.6				0.034
SMM (kg)	23.58 ± 1.6	24.06 ± 1.5	22.93 ± 1.3	22.38 ± 1.4	0.123	0.238	
Change	0.48 ± 0.3		−0.56 ± 0.4				0.049
BCM (kg)	28.10 ± 1.7	28.62 ± 1.7	27.38 ± 1.4	26.74 ± 1.5	0.123	0.195	
hange	0.52 ± 0.3		−0.64 ± 0.4				0.049
FM (kg)	13.54 ± 1.5	14.58 ± 1.6	17.73 ± 2.1	17.84 ± 2.4	0.059	0.844	
Change	1.03 ± 0.5		0.11 ± 0.9				0.148
2^nd^ cycle							
BW (kg)	61.62 ± 3.9	61.38 ± 3.2	59.10 ± 3.2	58.93 ± 3.3	0.917	0.866	
Change	−0.23 ± 1.0		−0.17 ± 1.1				0.945
FFM (kg)	44.72 ± 3.1	42.97 ± 2.2	43.31 ± 2.7	43.24 ± 2.3	0.116	1.000	
Change	−1.75 ± 1.0		−0.07 ± 1.3				0.445
SMM (kg)	23.98 ± 1.8	23.00 ± 1.4	23.16 ± 1.6	23.10 ± 1.3	0.080	0.933	
Change	−0.98 ± 0.5		−0.06 ± 0.7				0.234
BCM (kg)	28.52 ± 1.9	27.55 ± 1.5	27.61 ± 1.7	27.59 ± 1.4	0.138	0.86	
Change	−0.97 ± 0.6		−0.03 ± 0.8				0.295
FM (kg)	16.90 ± 2.5	18.42 ± 2.5	15.86 ± 3.0	15.54 ± 3.1	0.028	0.799	
Change	1.52 ± 0.3		−0.31 ± 1.1				0.234

Values are mean ± SE. Change = (8-week data) − (0-week data). *p^a^* values were derived from the Wilcoxon signed rank test between 0 weeks (baseline) and 8 weeks in the ONS group. *p^b^* values were derived from the Wilcoxon signed rank test between 0 weeks (baseline) and 8 weeks in the non-ONS group. *p^c^* values were derived from the Mann–Whitney test between the change value of the two groups. BW: body weight; FFM: fat-free mass; SMM: skeletal muscle mass; BCM: body cell mass; FM: fat mass.

**Table 3:** The changes of the European Organization for Research and Treatment of Cancer (EORTC) Quality of Life Questionnaire Core 30 (QLQ-C30) scores between groups.

Variable	ONS Group (*n* = 15)				Non-ONS Group (*n* = 19)					
	0w	8w	Δ	*p* ^a^	0w	8w	Δ	*p* ^b^	*p* ^c^	*p* ^d^
**Global health status/QoL**										
	55.6 ± 4.58	65.6 ± 4.06	10 ± 5.16	0.065	54.4 ± 4.66	59.2 ± 4.97	4.83 ± 4.29	0.323	0.929	0.477
**Functional scales**										
Physical	76.4 ± 4.06	83.6 ± 2.67	7.11 ± 4.34	0.154	75.8 ± 4.71	74.4 ± 3.92	−1.4 ± 3.55	0.825	0.847	0.306
Role	85.6 ± 5.11	92.2 ± 3.59	6.67 ± 6.67	0.286	82.5 ± 4.85	71.9 ± 6.25	−10.5 ± 7.45	0.171	0.699	0.158
Emotional	89.4 ± 2.75	88.9 ± 5.06	−0.56 ± 3.86	0.932	85.5 ± 4.12	81.6 ± 4.06	−3.95 ± 4.05	0.507	0.885	0.604
Cognitive	93.3 ± 2.72	91.1 ± 2.22	−2.22 ± 2.75	0.222	80.7 ± 4.28	81.6 ± 3.58	0.88 ± 4.32	0.906	0.026	0.677
Social	85.6 ± 4.26	88.9 ± 3.87	3.33 ± 4.93	0.603	91.2 ± 3.46	89.5 ± 5.13	−1.76 ± 4.75	0.461	0.215	0.306
**Symptom scales**										
Fatigue	37 ± 4.93	20.7 ± 4.58	−16.3 ± 7.1	0.041	33.3 ± 5.17	36.3 ± 5.69	2.93 ± 5.88	0.616	0.467	0.064
Nausea/Vomiting	8.9 ± 3.59	7.78 ± 3.2	−1.11 ± 4.14	0.705	13.2 ± 4.15	9.6 ± 3.45	−3.51 ± 3.01	0.221	0.480	0.512
Pain	4.45 ± 1.97	2.22 ± 1.51	−2.22 ± 2.22	0.317	26.3 ± 6.66	14.9 ± 4.92	−11.4 ± 5.71	0.039	0.006	0.178
Dyspnea	4.44 ± 3.03	11.1 ± 5.31	6.67 ± 3.56	0.102	15.8 ± 3.92	15.8 ± 4.68	0 ± 4.41	0.739	0.038	0.283
Insomnia	26.7 ± 7.42	26.7 ± 8.1	0 ± 9.2	0.722	24.6 ± 5	24.6 ± 7.14	0 ± 6.24	0.672	0.940	0.615
Anorexia	33.3 ± 5.63	17.8 ± 7.88	−15.6 ± 8.52	0.112	29.8 ± 7.16	24.6 ± 6.16	−5.26 ± 7.33	0.856	0.423	0.248
Constipation	11.1 ± 5.31	11.1 ± 4.2	0 ± 6.51	1.000	19.3 ± 6.41	12.3 ± 4.57	−7.02 ± 7.89	0.359	0.355	0.359
Diarrhea	4.44 ± 3.03	2.22 ± 2.22	−2.22 ± 3.94	0.564	12.3 ± 5.23	10.5 ± 5.13	−1.75 ± 4.01	0.655	0.313	0.944
Financial difficulties	33.3 ± 5.63	22.2 ± 5.31	−11.1 ± 7.03	0.154	35.1 ± 6.97	33.3 ± 5.7	−1.75 ± 6.97	0.668	0.955	0.284

Values are nean ± SE. Change (Δ) = (8-week data) − (0-week data). *p^a^* values were derived from the Wilcoxon signed rank test between 0 weeks (baseline) and 8 weeks in the ONS group. *p^b^* values were derived from the Wilcoxon signed rank test between 0 weeks (baseline) and 8 weeks in the non-ONS group. *p^c^* values were derived from the Mann–Whitney test between the 0-week values of the two groups. *p*^d^ values were derived from the Mann–Whitney test between the change values (Δ) of the two groups.

**Table 4:** Change of biochemical test between groups.

	Reference		ONS Group (*n* = 15)	Δ	Non-ONS Group (*n* = 19)	Δ	*p* ^a^
**Alb (g/dL)**	3.4~5.3	0w	4.19 ± 0.5	−0.39 ± 0.5	3.53 ± 0.1	0.26 ± 0.1	0.120
		8w	3.81 ± 0.1		3.79 ± 0.1*		
**T.P (g/dL)**	6.9~8.3	0w	6.91 ± 0.2	0.07 ± 0.1	6.56 ± 0.2	0.18 ± 0.2	0.391
		8w	6.98 ± 0.1		6.74 ± 0.1		
**Chol (mg/dL)**	<200	0w	145.46 ± 10.7	14.01 ± 10.4	148.11 ± 8.1	6.95 ± 8.7	0.945
		8w	159.47 ± 10.6		155.05 ± 6.7		
**ANC (10^3^/μL)**	2~9	0w	3.06 ± 0.4	0.69 ± 0.7	7.72 ± 2.9	−4.04 ± 3.0	0.430
		8w	3.76 ± 0.7		3.67 ± 0.6		
**TLC (10^3^/μL)**	1.5~8.0	0w	3.39 ± 1.5	−1.53 ± 1.5	2.94 ± 0.9	−1.40 ± 0.9	0.732
		8w	1.86 ± 0.2		1.54 ± 0.1		
**Hb (g/dL)**	12~16	0w	11.00 ± 0.4	−0.47 ± 0.2	11.02 ± 0.4	−0.27 ± 0.3	0.560
		8w	10.53 ± 0.4		10.75 ± 0.3		
**Hct (%)**	36~48	0w	33.49 ± 1.1	−1.09 ± 0.8	33.34 ± 1.2	−0.64 ± 0.8	0.706
		8w	32.40 ± 1.2		32.70 ± 0.9		

Values are mean ± SE. Δ (change) = (8-week data) − (0-week data). *p*^a^ values derived from the Mann–Whitney test between the ONS group and non-ONS group. * Wilcoxon signed rank test between 0 weeks (baseline) and 8 weeks (*p* < 0.05). Alb, albumin; T.P, total protein; Chol, cholesterol; ANC, absolute neutrophil count; TLC, total lymphocyte count; Hb, hemoglobin; Hct, hematocrit.

## Supplementary Materials

The following are available online at https://www.mdpi.com/2072-6643/11/5/1145/s1, Table S1: Cancer progression of ONS and non-ONS subjects over the study period (8 weeks), Table S2: Comparison of meal and snack intake between the ONS (*n* = 15) and non-ONS (*n* = 19) groups during the study period (8 weeks).
