# Supplementary material for: The Effect of Nutrition Intervention with Oral Nutritional Supplements on Pancreatic and Bile Duct Cancer Patients Undergoing Chemotherapy
Source: Nutrients. 2019 May 22;11(5):1145. doi: 10.3390/nu11051145 (PMC6566877; doi:10.3390/nu11051145)
Supplement: Supplementary file 1 [file nutrients-11-01145-s001.pdf]

Supplementary table S1. Cancer progression of ONS and Non-ONS subjects over the study period (8 weeks).

Cancer progression [a]	**ONS (n=15)**	**Non-ONS (n=19)**
Complete remission	0(0)	0(0)
Partial remission	2(13.3)	1(6.7)
Stable disease	10(66.7)	14(93.3)
Progressive disease	3(20.0)	4(26.7)

[a] n(%). All cancer progression represents the change of cancer volume before and after chemotherapy. *Complete remission* complete eradication of cancer; *Partial remission* decreased cancer volume to 50% or more; *Stable disease* decreased cancer volume to less than 50% or increased cancer to less than 120%; *Progressive disease* increased cancer volume to 125% or more despite chemotherapy.

Supplementary table S2 Comparison of meal and snack intake between the ONS (n=15) and Non-ONS (n=19) groups during the study period (8 weeks).

	**Variables**		**ONS (n=15)**	**Non-ONS (n=19)**	p[2)]
**Meal**	Energy (kcal)	0w	1224.20±88.3	1198.33±116.2	0.918
		8w	1311.57±82.5	1306.5±124.7	0.973
	Protein (g)	0w	50.27±6.6	55.38±6.1	0.607
		8w	65.98±5.5	70.39±8.14	0.918
	Carbohydrate (g)	0w	199.61±21.0	175.60±16.6	0.706
		8w	189.97±11.4	173.10±15.5	0.607
	Fat (g)	0w	32.70±4.7	32.31±4.5	0.784
		8w	32.90±4.6	37.65±5.2	0.706
**Snack (including ONS)**	Energy (kcal)	0w	263.87±48.3	273.87±53.9	0.973
		8w	649.32±41.7**	438.86±56.7*	0.006§§
	Protein (g)	0w	13.82±5.6	8.65±1.8	0.918
		8w	25.88±2.5**	13.58±1.8**	0.071
	Carbohydrate (g)	0w	48.31±9.4	45.96±7.6	0.918
		8w	105.67±6.4**	77.53±10.1	0.000§§§
	Fat (g)	0w	5.90±1.2	8.19±2.4	0.656
		8w	17.12±1.3*	11.03±1.6	0.000§§§

[1)] Mean±SE [2)] Statistical difference between ONS group and Non-ONS group derived from Mann-Whitney test (§: p <0.05, §§: p <0.01, §§§: p <0.001)

* Significant difference between baseline and 8w obtained by Wilcoxon signed rank test (*: p <0.05, **: p <0.01, ***: p <0.001)
